# The gut microbiome of solitary bees is mainly affected by pathogen assemblage and partially by land use

**DOI:** 10.1186/s40793-023-00494-w

**Published:** 2023-04-26

**Authors:** Gregorio Fernandez De Landa, Daniele Alberoni, Loredana Baffoni, Mateo Fernandez De Landa, Pablo Damian Revainera, Leonardo Pablo Porrini, Constanza Brasesco, Silvina Quintana, Francisco Zumpano, Martìn Javier Eguaras, Matias Daniel Maggi, Diana Di Gioia

**Affiliations:** 1 Facultad de Ciencias Exactas y Naturales, Centro de Asociación Simple CIC PBA, Instituto de Investigaciones en Producción Sanidad y Ambiente (IIPROSAM), Universidad Nacional de Mar del Plata, Centro Científico Tecnológico Mar del Plata, CONICET, Mar del Plata, Argentina; 2 Facultad de Ciencias Exactas y Naturales, Centro de Investigaciones en Abejas Sociales, Universidad Nacional de Mar del Plata, , Mar del Plata, Argentina; 3 Dipartimento di Scienze e Tecnologie Agro-Alimentari, University of Bologna, Viale Fanin 44, 40127 Bologna, Italy; 4 Facultad de Ciencias Exactas y Naturales, Instituto de Investigaciones Marinas y Costeras (IIMyC), Funes 3350, Universidad Nacional de Mar del Plata-CONICET, 7600 Mar del Plata, Argentina

**Keywords:** Xylocopa augusti, Eucera fervens, Halictidae, Gut microbiome, Nosema ceranae, Crithidia bombi, Nosema bombi

## Abstract

Pollinators, including solitary bees, are drastically declining worldwide. Among the factors contributing to this decline, bee pathogens and different land uses are of relevance. The link between the gut microbiome composition and host health has been recently studied for social pollinators (e.g. honeybees), whereas the information related to solitary bees is sparse. This work aimed at the characterization of the gut microbiome of the solitary bees *Xylocopa augusti*, *Eucera fervens* and *Lasioglossum* and attempted to correlate the gut microbial composition with the presence and load of different pathogens and land uses. Solitary bees were sampled in different sites (i.e. a farm, a natural reserve, and an urban plant nursery) showing different land uses. DNA was extracted from the gut, 16S rRNA gene amplified and sequenced. Eight pathogens, known for spillover from managed bees to wild ones, were quantified with qPCR. The results showed that the core microbiome profile of the three solitary bees significantly varied in the different species. *Pseudomonas* was found as the major core taxa in all solitary bees analyzed, whereas *Lactobacillus*, *Spiroplasma* and *Sodalis* were the second most abundant taxa in *X. augusti, E. fervens* and *Lasioglossum,* respectively. The main pathogens detected with qPCR were *Nosema ceranae*, *Nosema bombi* and *Crithidia bombi*, although differently abundant in the different bee species and sampling sites. Most microbial taxa did not show any correlation with the land use, apart from *Snodgrassella* and *Nocardioides,* showing higher abundances on less anthropized sites. Conversely, the pathogens species and load strongly affected the gut microbial composition, with *Bifidobacterium*, *Apibacter*, *Serratia*, *Snodgrassella* and *Sodalis* abundance that positively or negatively correlated with the detected pathogens load. Therefore, pathogens presence and load appear to be the main factor shaping the gut microbiome of solitary bees in Argentina.

**Supplementary Information:**

The online version contains supplementary material available at 10.1186/s40793-023-00494-w.

## Background

Pollinators represent a diverse and heterogeneous group of insects with an important role in the ecosystem, since they are responsible for the pollination of 75% of globally important crops [1]. Among pollinators, bees represent a monophyletic group, which include, besides the common honey bee (*Apis mellifera*), more than 20.000 species belonging to different genera [2]. Differently from most insects, bee biodiversity peaks in temperate areas but very few are present in the tropical ones [3]. Often, non-*Apis* bees are more effective pollinators than honey bees for both wild flora and crops [4, 5]. The contribution of wild pollinators should not be underestimated because they can fully pollinate crops without the presence of any kind of managed pollinators [6, 7] although it was also demonstrated that the highest efficiency in crop pollination is achieved when wild pollinators and managed bees are co-habiting the same ecological niche [8]. The Buenos Aires province, in Argentina, is one of the most important areas for farming [9] and Ramello et al. [10] found up to 90 different species of wild bees in that province. Among them, *Xylocopa augusti*, *Eucera fervens* and bees of the Halictidae family are known for their role as wild pollinators. The *Xylocopa* genus (Xylocopinae: Xylocopini) includes more than 470 species all over the world [2], and *X. augusti* is one of the most prevalent in South America [11, 12]. In contrast to other bees, *X. augusti* can use buzz-pollination visiting a wide range of plant species to obtain pollen and nectar [13–15]. Bees from the family (Hymenoptera: Halictidae) are small with a cosmopolitan distribution and over 4.000 species have been described worldwide [15]. Particularly, *Lasioglossum* and *Haictillus* genera group some important native wildflowers visitors in Argentina [16] with a solitary behavior. Ramello et al., [10] found up to 21 different species of halictids, nevertheless the identification at genus and species level is arduous with morphometric tools posing a taxonomical issue in the research activities [17–19]. Finally, Buenos Aires province also harbors wild bees belonging to the Eucerini tribe (with more than 780 species distributed worldwide) [2] with importance for the agricultural systems, such as the species *Eucera fervens* [20] (formerly known as *Peponapis fervens*, subgenera Peponapis [21]). Bees belonging to the Peponapis subgenera are native from South America [22, 23], and usually their pollination activity rely mainly upon plants of the genus *Cucurbita* [24] and especially on pumpkins [10].

It is known that pollinators are drastically declining worldwide [25, 26]. Different factors are contributing to this decline, like habitat loss, climate change, urbanization, agricultural intensification, pathogens spread at global level [27–29], all complex variables that may act in synergy [30]. Bee pathogens show lethal and sublethal effects on bees generally reducing their lifespan [31] and among pathogens, parasites have been identified as main drivers of wild bees decline [32, 33]. Recent works have focused on the relationship between landscape and bee pathogens, and especially vegetation cover (with different degrees of biodiversity) was reported as a determining factor in the parasites and pathogens spread and dynamics [34].

Commensal microbial communities present in bees gut were recently positively correlated with the host growth and health [35, 36]. In healthy bees, microbial communities are carrying out fundamental functions, such as the support in nutrient acquisition, the regulation of immune responses or defense against pathogens and parasites [37, 38], and detoxification of xenobiotics mainly deriving from agriculture [39]. Changes in the gut microbiome composition may have negative consequences in the host, affecting bees’ health and fitness [40]. Most of the bee microbiome studies regard *A. mellifera* species and little information are available on wild pollinators. Moreover, little is known about the relationship between landscape, pathogens, and bee gut microbial communities. Recently published research reported that both internal and external pathogens are drives the gut microbial community [38, 41], but also environment can deeply shape the gut microbiome [42].

In this study three solitary wild bee taxa, *X. augusti, E. fervens* and *Lasioglossum,* with an important role in crop pollination in the Buenos Aires Province, were selected to investigate the gut microbiome composition and the influence of land use and pathogens on the gut microbiome. The questions on which this study is focused are: *(i)* does the gut microbiome vary in the different bee taxa?; *(ii)* are there variations in the distribution and amount of the different bee pathogens in the studied bee species?; *(iii)* what is the effect of land use on the microbiome composition and biotic stresses distribution? *(iv)* are the core gut microorganisms and the pathogens load related?

## Methodology

### Study sites

For this study, bee capture sampling was carried out in 3 different sites characterized by different land use and landscape. The first site was located in Santa Paula’s Farm (SP) and it is specialized on Kiwi fruit and Cucurbitaceae vegetables production (route 226, km 10, Mar del Plata, Buenos Aires; 37° 56´ 0.69´´ S; 57° 40´ 40.53´´ W), the second selected site was a Natural Reserve of Buenos Aires Province, Reserva Natural Paititi (RP), with native grasslands and organic agriculture (37° 54′ 47.774'' S, 57° 48′ 44.806'' W), and, finally, a sampling site was a plant nursery, Vivero Antoniucci (VA), with flowering garden bushes and trees (38° 1′ 42.014''S, 57° 37′ 59.374'' W). In all the sapling sites a wide variety of bee species were present including the domestic bee *Apis mellifera*.

### Spatial analysis

**Fig. 1:**
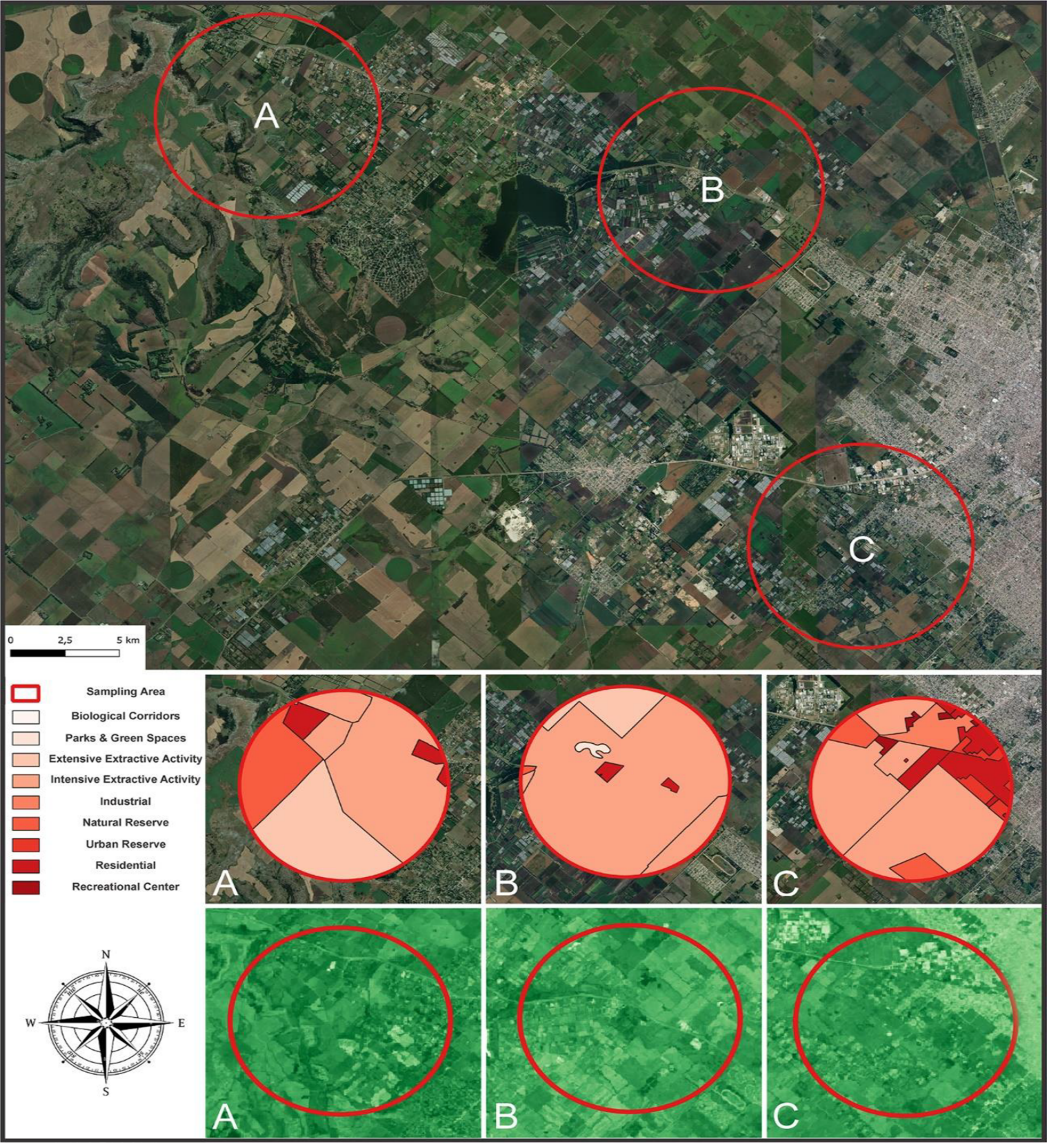
Spatial Characterization. The figure on the top represents the three sampling sites (A = *Reserva Natural Reserva Natural Paititi*; B = Santa Paula; C = *Vivero Antoniucci*). The three figures in the middle with different shades of red shows the different land uses used in the area with the respective references on the left according to the Argentinian national database on land use. Finally, the three figures on the bottom shows the map constructed from the NDVI values, the shades of green correspond to: dark green correspond to a high absorbance of UV light (healthy vegetation cover); light green correspond to lower absorbance (tilled soil, buildings, and roads)

For the spatial characterization of the sampling zones, satellite images from the Landsat 8 satellite were used. These images were taken from the Google Earth Engine repository, with atmospheric corrections made under the name "'LANDSAT/LC08/C01/T1_TOA'". Using the Google Earth Engine platform, the free-access satellite images corresponding to the dates on which the sampling was carried out were downloaded. Only those images with a cloud cover of less than 20% were considered. From the images obtained, a representative image of the median of the filtered images was constructed, thus making it possible to work with a single image containing information from the entire sampling period. This image was exported and processed on the freely available software QGIS (https://qgis.org/) where the normalized difference between the infrared and red bands was calculated, an operation that gives a result known as NDVI, which indicates the predominance or not of vegetation by assigning pixel values between 0 and 1. The NDVI index is considered as a good indicator of the physical properties of the vegetation cover such as, leaf area index, vegetation condition and biomass [43]. To complete the spatial characterization, information on land use available in the repository of the Geographic Information System of the province of Buenos Aires "urbasig" (https://www.urbasig.gob.gba.gob.ar) were used. Three points corresponding to the three sampling zones were added to the final images in QGIS, from which a 3 km radius buffer was constructed according to the foraging range of the different species. Figure 1 summarizes the images detailed above.

### Experimental design

The work includes the spatial analysis of three different sites, the sampling of solitary pollinators in the study sites, the characterization of the pollinator microbiome and the detection, in each sampled individual of 10 different pathogens known to be dispersed by social bees to solitary pollinators. These data are collected in order to correlate the growing anthropization with a greater presence of pathogens and a consequent imbalance of the microbiota.

### Bee sampling and DNA extraction

Different collection campaigns were carried out in each site in the period between December and April of the years 2019 and 2020 (corresponding to the Spring–Summer-Autumn season). For each collection sites, three transects of 70 m were delimited, then the collection was performed in a time interval of 30 min per transect, during sunny days between 9 a.m and 1 p.m. Bees were captured directly from flowers with the use of a homemade bee vacuum.

In total 56 bees were collected, kept separately in plastic vials and maintained at -20 ºC until further analysis. Bees identification through morphometric analysis on the head, wings, body structure and genitals was carried out under a stereo microscope with a × 40 magnification. Total genomic DNA was individually extracted from sampled guts using the High Pure PCR Template Preparation kit (Roche Diagnostics) according to [44]. Extracted DNA was used for bees identification through COI (Cytochrome Oxidase subunit I) gene amplification and sequencing, microbiome study and pathogens analyses. COI amplification and Sanger sequencing of amplified amplicons has been performed following the same protocol of Fernandez de Landa et al. [20].

### Pathogen identification and quantification

The following pathogens, considered to be the most common for spill-over between different bee species co-populating the study area, were analyzed through qPCR (StepOne™ Real-Time PCR System, Applied Biosystems) according to [44] relaying on gut extracted DNA: *N. ceranae, N. apis, N. bombi, Crithidia bombi, Lotmaria passim, Apicystis bombi, Ascosphaera* spp. and *Apis mellifera* Filamentous Virus (AmFV)*.* All qPCR reactions were performed with qPCRBIO SyGreen Mix Hi-ROX (Ca. number PB20.12–20, PCRBIO systems, London, UK), following the manufacturer’s instructions. DNA samples were diluted 1:10 prior to loading in the reaction well. The specific primers used, the melting temperature and the reaction efficiency are reported in Additional file 1: Table S1. Standard curves were constructed using PCR products of the target amplicon gene. The PCR products were purified, quantified with Qubit™ dsDNA HS Assay Kit (Thermo Fisher, Milan, Italy) and the number of copies was determined based on the amplicon length and total concentration (ng/µl). Amplicons were serially diluted to obtain standards ranging from 10^4^ to 10^8^ gene copies. qPCR data output was standardized for reaction efficiency and melting temperature was verified for every sample and target. To better compare the intensity of each pathogen infecting solitary bees, only bees with a positive C_t_ value showed by qPCR analysis were considered for further analysis. Finally, according to [44], obtained raw data for the analyzed Nosema species, were corrected by copy number. For *N. ceranae* and *N. bombi* data are expressed as *N. ceranae* Units (NcU) and *N. bombi* Units (NbU).

### Gut microbiome and bioinformatic analysis

The microbiome analysis on sampled bees was performed using NGS technology on an Illumina MiSeq platform in 300 PE and based on the 16S *rRNA* gene, regions V5-V7. Libraries were prepared according to [45] with some variations: KAPA Hi-Fi PCR Master Mix (Roche diagnostics) was used to amplify the target DNA with a maximum of 25 PCR cycles. The primers used, reported in Additional file 1: Table S1, allow the differentiation of amplicons deriving from bacteria (about 470 bp) from that deriving from plants (in pollen) plastids (about 720 bp) according to [46].

In order to exclude the amplified 720 bp band, the obtained libraries were purified with the precast E-Gel Size Select II 2% gel (Cat. Number G661012, ThermoFisher, Milan, Italy) loaded on E-Gel™ Power Snap Electrophoresis Device (ThermoFisher, Milan, Italy). Over the purify products a new PCR was performed in order to prepare the libraries with the Nextera XT DNA Library Preparation Kit (Cat. Number FC-131–2004, Illumina, Milan, Italy). The PCR products were purified in this case using magnetic beads (AMPure kit by Beckman Coulter). The purified libraries composed of 470 bp amplicons were quantified using Qubit 2.0 Fluorometer (Thermo Fisher Scientific, Milan, Italy) and the final libraries pool prepared for the sequencing at Macrogen Sequencing facilities (Seoul, South Korea). Raw sequences obtained from Macrogen were analyzed with QIIME ver. 1.9.1 [47] separating the sequencing according to the identified insect genus. R1 and R2 sequences were joined with join_paired_ends.py, allowing a minimum overlap of 5 nucleotides. Chimeras were detected with Usearch61 [48] and identified chimeras eliminated from the file. Clustering into operational taxonomic units (OTUs) (97% identity) was performed and representative OTUs were assigned using the most updated SILVA database v.136. The phylogenetic tree was generated using make_phylogeny.py (fasttree). Diversity analyses were performed with the script core_diversity_analysis.py. α–Diversity was evaluated using Chao1, Observed OTU e PD whole tree metrics; β–diversity was evaluated using both weighted and unweighted UniFrac. The obtained rarefied biom table was then used to provide information of taxonomic groups within each sample at all taxonomic levels as relative abundances. OTUs having less than 0.1% abundance were removed.

### Statistics

Linear Models (LMs) have been used to compare the pathogens loads among species and among sites. The responses variables were the counts of *N. bombi*, *N. ceranae* and *C. bombi*. In all models, the following effects were testes: species (categorical variable: *Lasioglossum*, *E. fervens* and *X. augusti*), and sampling sites (categorical variable: Reserva Natural Paititi (RP), Santa Paula’s farm (SP), and Vivero Antoniucci (VA)).

To compare the gut microbiota of the different solitary bees and the effect of land use on gut microbiota composition we used univariate and multivariate analysis, respectively. To perform univariate analysis, each genus of the gut microbiota with at least 1% of relative abundance was tested for normality (Shapiro–Wilk test) and homogeneity of variance (Levene test). Depending on the assumptions of each genus, univariate comparisons were performed using ANOVA or Kruskal–Wallis followed by Tuckey test or Dunn test, respectively. Pairwise multiple comparisons were performed using Permutational multivariate analyses of variance (PERMANOVA), based on Bray–Curtis distances and 9999 permutations. All the variables included in the PERMANOVA assumed the assumption of homogeneity. A nonmetric multidimensional scaling (nMDS) based on Bray–Curtis distances and 20 minimum and 200 maximum random starts was also performed to corroborate the obtained results in PERMANOVA analysis [49]. These analyses were performed using ‘vegan’ R package version 4.2.1 [50, 51]. In order to analyze the relation between the pathogens and the gut microbiota, a Spearman correlation between each pathogen and microbiota genus was calculated. Finally, PCA analysis was performed using packages FactoMineR and factoextra, taking into consideration 21 taxa at species and/or genus level. All tests were two-tailed with a significance level of *P* ≤ 0.05.

## Results

### The taxonomical identification of solitary bees

The 56 wild bees collected in this work belonged to 3 different genera (Additional file 1: Table S2). 30 individuals were identified as *Lasioglossum* based on morphometric analysis with stereo microscope on the head, wings, body structure and genitals (10 individuals for each study site). The COI sequence of *Lasioglossum* individuals did not match with the sequences deposited the NCBI GenBank, therefore it was not possible to further discriminate at species level. However, the collected bees had the same morphological traits therefore they are belonging to the same species. A total of 11 individuals were identified as *Xylocopa augusti* (4 individuals from VA, 5 individuals from SP and 2 individuals from RP). Finally, 15 individuals were identified as *Eucera fervens* (10 individuals from SP and 5 from RP). The limited number of *X. augusti* and *E. fervens* individuals was due to the limited number of bees that was possible to recover from the collection areas at the time of sampling. In all sampling sites *A. mellifera* and *Bombus* spp. were abundantly present, although not included in the study.

### The spatial characterization of the sampling sites

The 3 chosen study sites varied regarding the land use and the human impact. The RP site was considered as the area with the lowest level of human impact, considering that more than 18% of its territory is a natural reserve characterized by abundant native flora where the bees were collected. On the contrary, the area with the highest anthropic impact was SP with most of its surface (91.5%) dedicated to intensive farming. Finally, VA presents 15% of high-density residential areas, 4% of urban reserves (biological corridors), and more than 75% of intensive farming. No differences were observed regarding NVDI analysis (a) RP (NDVI mean = 0.57); (b) SP (NDVI mean = 0.49); (c) and VA (NDVI mean = 0.50). Land characterization and proportion of land use are reported in Additional file 1: Table S3.

### The identified pathogens in the solitary bees gut and their spatial distribution in the sampling sites

All the collected wild bees were found uninfected by *A. bombi, Ascosphaera* spp., *L. passim, N. apis,* and *Am*FV when analyzed in PCR. On the contrary, the pathogens *N. bombi, N. ceranae* and *C. bombi* were detected and quantified with qPCR.

**Fig. 2:**
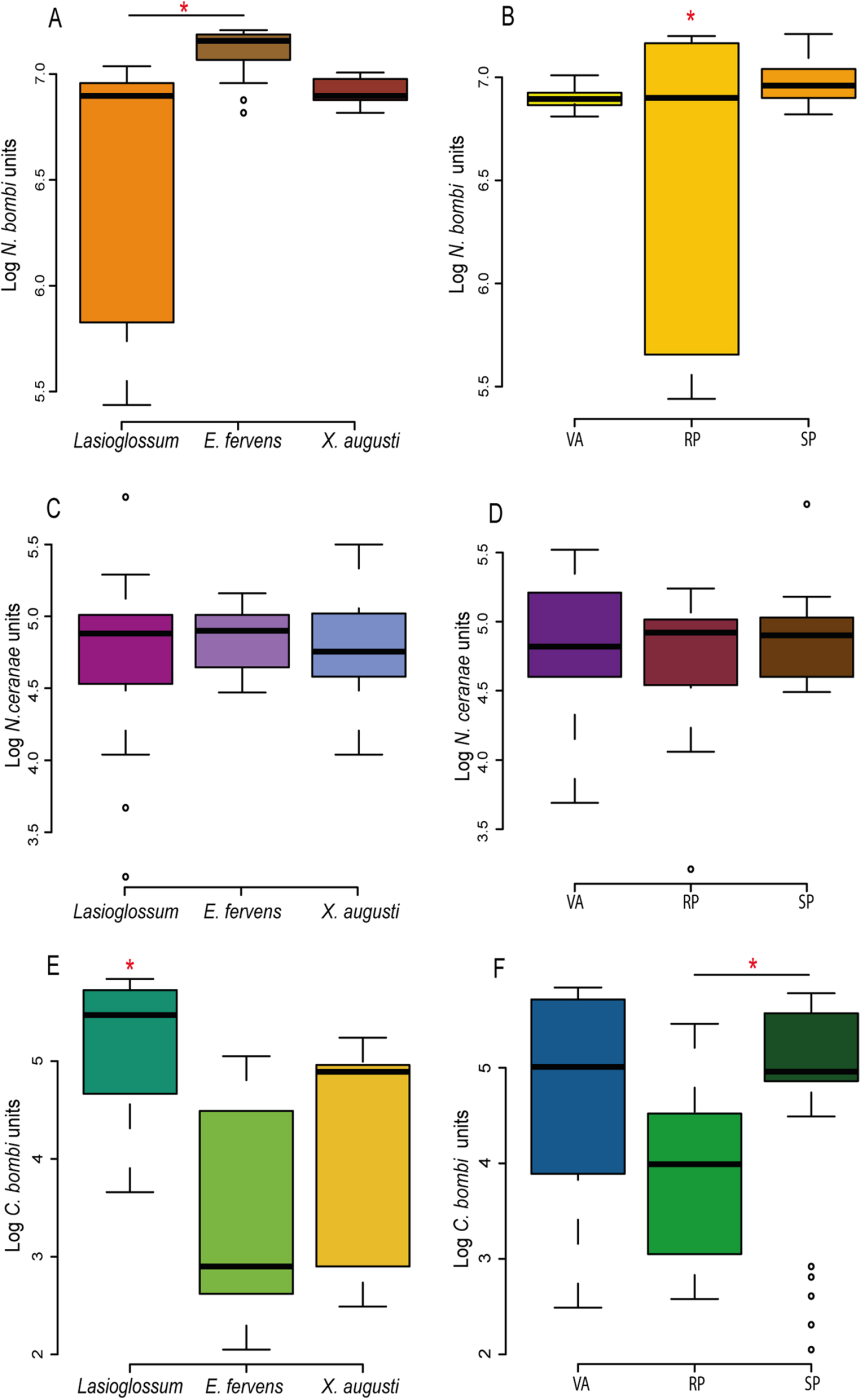
Boxplot describing the total pathogens count regarding the solitary bee species and sampling site analyzed in this work. **A** qPCR results for Nosema bombi expressed in units per bee; **B** qPCR results for Nosema bombi expressed in units per sampling site; **C** qPCR results for Nosema ceranae expressed in units per bee; **D** qPCR results for Nosema ceranae expressed in units per sampling site; **E** qPCR results for Critidia bombi expressed in units per bee; **F** qPCR results for Critidia bombi expressed in units per sampling site; The sampling sites: *VA* Vivero Antoniucci; *RP* Reserva Paititi; *SP* Santa Paula Farm. * p

*N. bombi* average counts in sampled species was Log 6.57, 7.1 and 4.78 *N. bombi* units (NbU) for *Lasioglossum*, *E. fervens* and *X. augusti*, respectively Fig. 2A, Additional file 1: Fig. S1A–S1C). NbU were significantly different when comparing *Lasioglossum versus E. fervens* (*p* < 0.001), whereas the comparison of *E. fervens versus X. augusti* and *X. augusti versus Lasioglossum*, was non-significant (*p* = 0.453 and *p* = 0.198 respectively). The intensity of *N. bombi* varied among sites with different land uses (Fig. 2B). While the intensity of *N. bombi* detected SP *versus* VA did not show significant differences, *N. bombi* intensity level in RP was significantly lower when compared to SP and VA (*p* < 0.05).

The level of *N. ceranae* was not significantly different among different bee speces and the different sampling sites (Fig. 2C–D; *p* > 0.05) where average counts of Log 3.32, 3.14 and 3.71 *N. ceranae* units (NcU) were obtained for *Lasioglossum*, *E. fervens* and *X. augusti* respectively (Additional file 1: Fig. S1D–F)). *Nosema ceranae* count, in contrast to *N. bombi,* did not vary within the study sites.

The average counts of *C. bombi* varied among wild bee species, with values of Log 5.16, Log 3.35 and Log 4.27 *C. bombi* units (CbU) for *Lasioglossum*, *E. fervens* and *X. augusti*, respectively (Fig. 2E; Additional file 1: Fig. S1G–I). CbU were significantly higher in *Lasioglossum* with respect to *E. fervens* and *X. augusti* (*p* = 0.001 and 0.043 respectively), although the comparison of *X. augusti vs E. fervens* was not significant (*p* = 0.11). Moreover, *C. bombi* intensity did not vary comparing SP *vs* VA (*p* > 0.05) and VA *vs* RP (*p* > 0.05), whereas significant differences were observed between SP and RP (*p* < 0.01; Fig. 2F) with the latter showing the lowest intensity levels of *C. bombi.*

### A simple gut microbiome characterizes solitary bees

A total of 56 samples (30 samples for *Lasioglossum*, 15 samples for *E. fervens* and 11 for *X. augusti*) were subjected to NGS analysis. About 11.4 million raw reads were obtained from the sequencing (7.6 million reads for *Lasioglossum,* 2.33 million reads for *E. fervens,* 1.4 million reads for *X. augusti*). A total of 7 samples for *Lasioglossum* 4 samples of *E. fervens* and 2 samples of *X. augusti* were excluded from the downstream analysis due to the low number of reads (< 36,000). Therefore, within the 43 remained samples, a total of 5.9 million reads passed the quality control and the chimera check analysis with an average of 83 k joint reads per sample for *Lasioglossum* 57 k joint reads per sample for *E. fervens,* 52 k joint reads per sample for *X. augusti*. The NGS data at phylum, family and genus levels are deposited in Mendeley repository (see section “Data Availability”).

The α-diversity indexes Chao1, Observed OTUs and PD Whole Tree did not show significant variations among sampling sites (Additional file 1: Table S4). On the contrary β-diversity indexes resulted significant comparing *E. fervens* and *X. augusti* microbiota among different sites for both Weighted and Unweighted Unifrac, whereas *Lassioglossum* gut microbial diversity among sites was not significant (data reported in Additional file 1: Table S5).

#### The gut microbiome of Lasioglossum

The gut microbiome of *Lasioglossum* at phylum level is mainly composed of Proteobacteria (89.44%), with low percentages of Firmicutes (4.95%), Actinobacteria (1.02%), Bacteroidetes (0.48%) and Other_taxa (4.11%). Within Proteobacteria, Pseudomonadaceae and Enterobacteriaceae were the most abundant bacterial families with a relative abundance of 39.86% and 33.11%, respectively, whereas Neisseriaceae and Anaplasmataceae were present at 5.15% and 3.66%, respectively. Within the same phylum, other frequently found taxa were Rhizobiaceae (1.79%), Burkholderiaceae (1.34%), Orbaceae (1.12%), Sphingomonadaceae (0.97%), Caulobacteraceae (0.57%), and Rhodocyclaceae (0.45%). The major represented family within Firmicutes was Lactobacillaceae (4.48%) whereas whithin Actinobacteria was Bifidobacteriaceae (0.3%).

At genus level, *Lasioglossum* gut microbiome hosts two prevalent microbial genera: *Pseudomonas* and *Sodalis* with a relative abundance of 39.84% and 30.16%, respectively. Other prevalent taxa are *Lactobacillus* and *Wolbachia* (4.47% and 3.65%, relative abundance respectively). Notably, *Snodgrassella* was found in only one sample, but with a relative abundance of 69.33%. Other detected microbial genera, although present at a lower percentage, are *Rhizobium* (1.75%), *Enterobacter* (1.45%), *Gilliamella* (1.07%) and *Sphingobium* (0.76%). Finally, minor microbial taxa with a relative abundance below 1%, when grouped reach an average relative abundance of 8.13%. Prevalence values (number of colonized individuals with a target microbial taxon divided by the total analyzed individuals) results of 100%, 85.75%, 78.57 and 50% respectively for *Pseudomonas*, *Sodalis*, *Wholbachia* and *Lactobacillus*.

#### The gut microbiome of E. fervens

*E. fervens* gut microbiome is composed of 4 major phyla, Proteobacteria (72.18%), Tenericutes (15.82%), Firmicutes (3.36%) and Actinobacteria (2.11%). At family level, two major core families were detected: Pseudomonadaceae (Proteobacteria phylum) and Spiroplasmataceae (Tenericutes phylum) with 56% and 17.40% relative abundance, respectively. Less abundant, although present at relevant percentages, Enterobacteriaceae (7.19%), Orbaceae (6.12%), and Burkholderiaceae (2.48%) were detected within Proteobacteria. Lactobacillaceae families were detected with an average relative abundance of 2.40%. Other families with more than 1% relative abundance detected in *E. fervens* gut were Anaplasmataceae (1.66%), Neisseriaceae (1.23%) and Rhodocyclaceae (1.82%). Moreover, Microbacteriaceae belonging to the Actinobacteria phylum, were detected at 1.10%. Minor microbial taxa with a relative abundance below 1%, when grouped reached an average relative abundance of 9.81%.

In agreement with the highest taxonomy level, at genus level two main microbial genera were detected in *E. ferverns*: *Pseudomonas*, with an average relative abundance of 50.91% followed by *Spiroplasma* with 15.82% average relative abundance. *Gilliamella*, *Serratia* and *Lactobacillus* were also prevalent and show an average relative abundance of 5.41%, 2.28% and 2.18%, respectively. Other less abundant genera were *Acidovorax* (1.28%), *Sodalis* (1.17%), *Weissella* (0.64%), *Nocardioides* (0.56%) and *Pantoea* (0.29%). Calculated prevalence was of 100% for *Pseudomonas*, 45% for *Spiroplasma*, 54.54% for *Gilliamella*, 72.72% for *Serratia* and 63.63% for *Lactobacillus*.

#### The gut microbiome of X. augusti

The most abundant phyla present in *X. augusti* gut microbiome were Proteobacteria (49.01% average relative abundance), Firmicutes (35.04%), Actinobacteria (4.70%), Bacteroidetes (4.06%) and Tenericutes (1.72%). As observed for both *E. fervens* and *Lasioglossum,* Pseudomonadaceae was the most abundant bacterial family with an average relative abundance of 39.99%. However, in the case of *Xylocopa*, Lactobacillaceae was the second major taxa with 24.56% average relative abundance. Enterobacteriaceae, Bifidobacteriaceae and Weeksellaceae showed a relative abundance of 5.37%, 3.67% and 3.55%, respectively. Microbacteriaceae (1.11%), Anaplasmataceae (2.08%), Rhodocyclaceae (2.02%), Spiroplasmataceae (1.94%), and Streptococcaceae (2.06%) showed a low prevalence and were occasionally found with an average relative abundance above 1%. Minor microbial taxa with a relative abundance below the 1%, when grouped reached an average relative abundance of 10.11%.

In *X. augusti* the most abundant microbial genera were *Pseudomonas* and *Lactobacillus* with an average relative abundance of 36.05% and 30.56%, respectively and a prevalence of 100% and 88.89% respectively. These genera were detected in the majority of *X. augusti* samples, showing a high frequency of colonization. Less abundant taxa although frequently found were *Apibacter* and *Bifidobacterium* that accounted for an average amount of 3.05% and 3.17%, respectively, and with a prevalence of 44.45% and 55.55% respectively. The genus *Wolbachia* (average 1.88%), *Spiroplasma* (1.49%), *Serratia* (1.69%), *Lactococcus* (1.48%), *Pantoea* (1.33%) and *Methyloversatilis* (1.08%) are occasionally found with a relative abundance above 1%. Finally, a very large number of microbial taxa with a relative abundance lower than 1% were found in the *X. augusti* gut, grouped in “other” with a total relative abundance of 11.6%.

### The comparison of the different microbiome profiles within solitary bees shows shared microbial taxa and some peculiar genera

In all tested solitary bees the most represented and co-shared genus was *Pseudomonas*, however several differences were observed in pairwise comparisons. The *p* values obtained from these comparisons are summarized in Table 1. The genera *Acidovorax, Geothermobacter, Gilliamella, Methyloversatilis, Nocardioides, Panotea, Serratia, Spiroplasma* and the group “Other” were significantly more abundant in *E. fervens* compared to *Lasioglossum* (*p*<0.05)*. Bifidobacterium, Brevundimonas, Chryseobacterium, Massilia, Rhizobium* and *Sphingobium* abundances were higher in *Lasioglossum* compared to the other bees (*p*= 0.001).

**Table 1:**
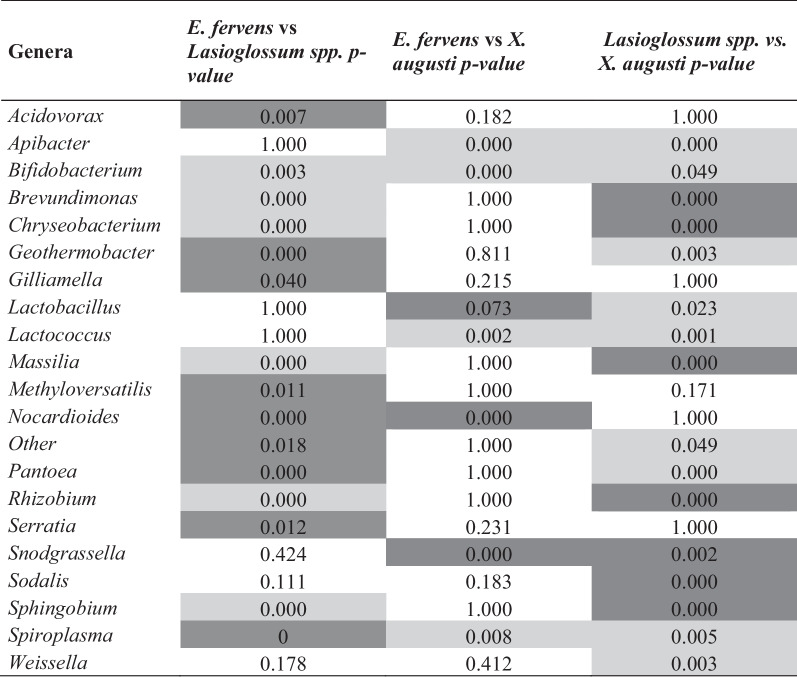
Pairwise comparison of bacterial relative abundance in the bee genus

The table summarizes the pairwise comparisons between pollinators species studied in this work, for the relative abundance of each microbial genus

The *p*-values of the pairwise comparisons are shown

In the pairwise comparison the dark gray means that the first named bee genus has higher relative abundance than the second, whereas the light gray indicates the opposite, the second named bee genus has higher relative abundance than the first. Only comparisons with p-values < 0.05 were considered as significant

**Fig. 3:**
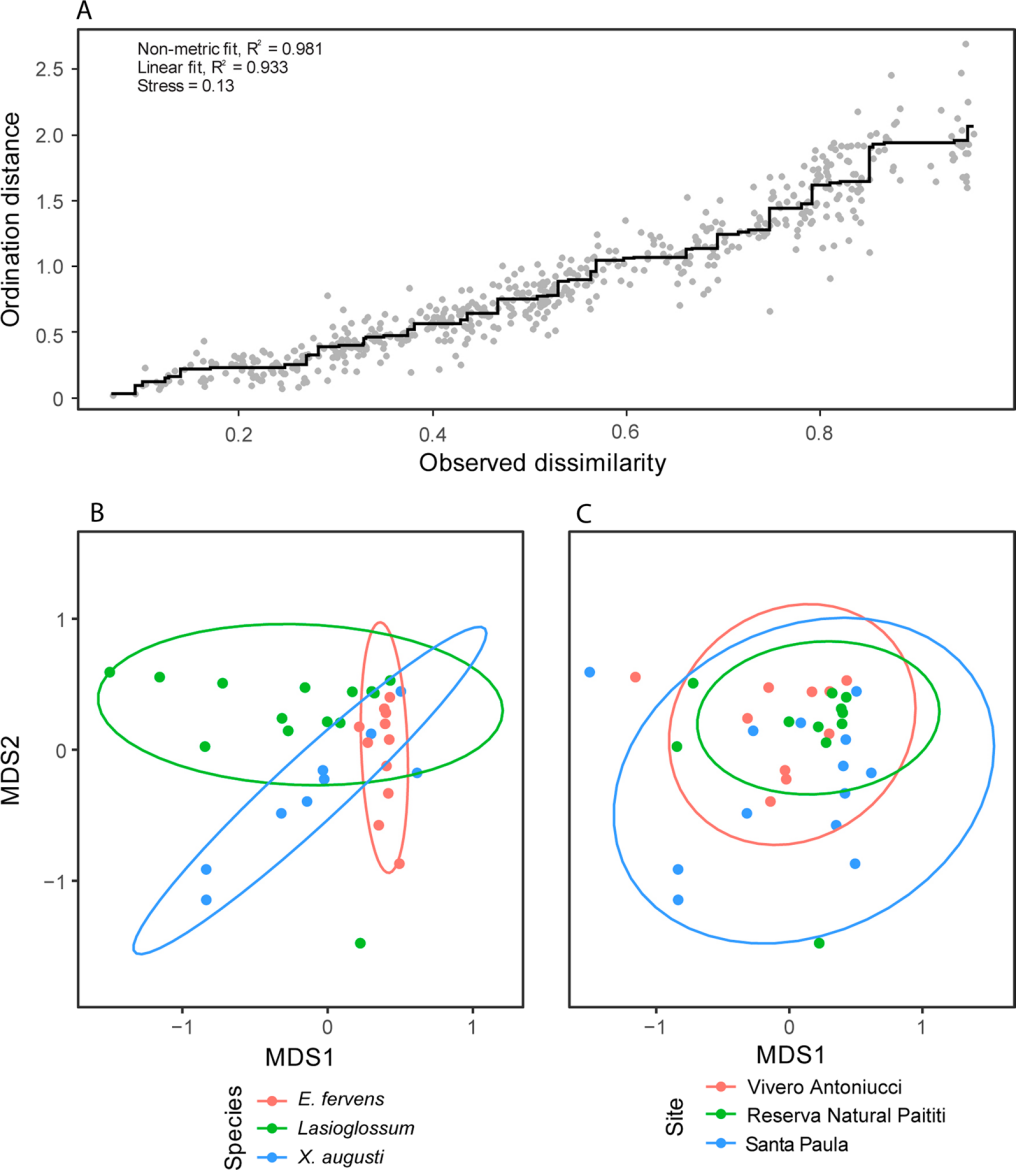
**A**–**C** Gut bacteria assemblage NMDS. Non-metric multi-dimensional scaling (NMDS) for the gut bacteria assemblage. Each points represent individuals. **A** Non-metric and linear fit (R2) and stress level between the ordination distance and the observed dissimilarity, indicating the analyses were accurate. **B** The bacterial assemblage varied within species **C** but no differences were observed in the bacterial assemblage at locality scale

Significant differences were found comparing gut bacteria relative abundance of *E. fervens versus X. augusti*. *Apibacter, Bifidobacterium, Lactococcus* and *Spiroplasma* were significantly higher in *X. augusti* (*p* < 0.05) whereas *Lactobacillus, Nocardioides* and *Snodgrassella* showed higher relative abundance in *E. fervens* (*p* < 0.05). The remaining bacterial genera did not present significant differences. Comparing the gut microbial groups relative abundance between *X. augusti* and *Lasioglossum*, the genera *Apibacter, Bifidobacterium, Geothermobacter, Lactobacillus, Lactococcus, Panotea, Spiroplasma* and *Weissella,* presented significant higher relative abundance in *X. augusti* (all *p* < 0.05). On the contrary, *Brevundimonas, Chryseobacterium, Massilia, Rhizobium, Snodgrassella, Sodalis* and *Sphingobium* genera showed significant higher relative abundance in *Lasioglossum* (all *p* < 0.05). Finally, PERMANOVA analysis (F = 6.20, *p* < 0.001) revealed significant differences for all pairwise comparisons (all *p* < 0.01). The NMDS analysis presented good non-metric and linear fit to the observed dissimilarity, and a fair stress level (Fig. 3A) and showed a relative segregation between species, with an area of overlap close to the origin of the axes (*p* < 0.05, Fig. 3B).

### The land use partially affects the gut microbiome of the solitary bees

The 22 most abundant microbial genera populating the gut microbiome of the three solitary bees were considered for the correlation analysis with the environment. Among these genera, 20 did not show any statistical difference among the different study sites in all solitary bees (*p* > 0.05). On the contrary *Snodgrassella* and *Nocardioides* recorded a higher relative abundance in RP when compared to the VA (*p* < 0.001 and *p* < 0.05, respectively). Also, while both genera were equally present in SP and VA sampling sites (*p* = 1.000 and *p* = 0.514, respectively), *Snodgrasella* showed higher abundance in RP if compared to SP (*p* = 0.011). This difference was not observed for *Nocardioides* (*p* = 0.304). Regarding the comparisons of each bacterial taxon among the analyzed species, two genera displayed a similar relative abundance in all pollinator species: *Pseudomonas* (*p* = 0.306) and *Wolbachia* (*p* = 0.491). PERMANOVA analysis showed differences between land uses (F = 2.02, *p* < 0.05), however, in pairwise comparisons, after Bonferroni correction, no effect of the different land uses was observed on gut microbiome composition (*p* > 0.05). The NMDS analysis showed a high overlap among sampling sites (Fig. 3C). Even when the Bonferroni correction shows that there is no significant effect of the land use over the gut microbiome, the preliminar obtanined differences of the PERMANOVA reflects a partial tendency illustrated on Fig. 3C where the size of the circle is positively correlated with diversity of the bacterial genus.

### The bee pathogens are shaping the gut microbiome of solitary bees

**Fig. 4:**
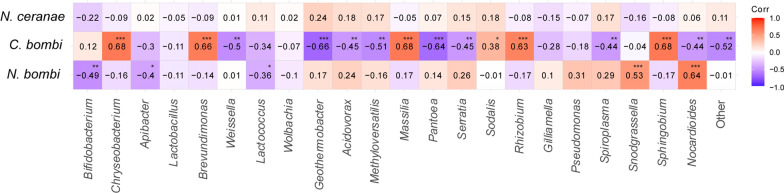
Spearman heat-map for pathogens and intestinal bacteria correlation. Heat-map for Spearman's correlation indices. Positive values (in red scales) represent positive correlation between bacterial genera and pathogen, while negative values (in blue scale) represent a negative correlation. * *p* ≤ 0.05; ** *p* ≤ 0.01; *** *p* ≤ 0.001

The three pathogens detected showed different levels of correlation with the microbial taxa populating the gut microbiome of the analyzed solitary bees (Fig. 4). While *N. ceranae* did not show relevant correlation with the gut microbial taxa described (*p* > 0.05), *N. bombi* and *C. bombi* strongly influenced the microbiome profile positively or negatively correlating with multiple microbial taxa. On the one hand, *N. bombi* was negatively correlated with *Bifidobacterium, Apibacter* and *Lactococcus* (*p* < 0.05)*,* whereas it was positively correlated with *Snodgrassella* and *Nocardioides* (*p* < 0.05)*. C. bombi* positively correlated with *Rizobium*, *Sphingobium, Massilia, Brevundimonas* (*p* < 0.01) and *Sodalis* (*p* < 0.05) whereas it negatively correlated with *Pantoea*, *Spiroplasma*, *Serratia*, *Acidovorax* and other minor microbial taxa (*p* < 0.01) (Fig. 4).

### An integrate effect of pathogens, land use and bee species over the gut microbiome

**Fig. 5:**
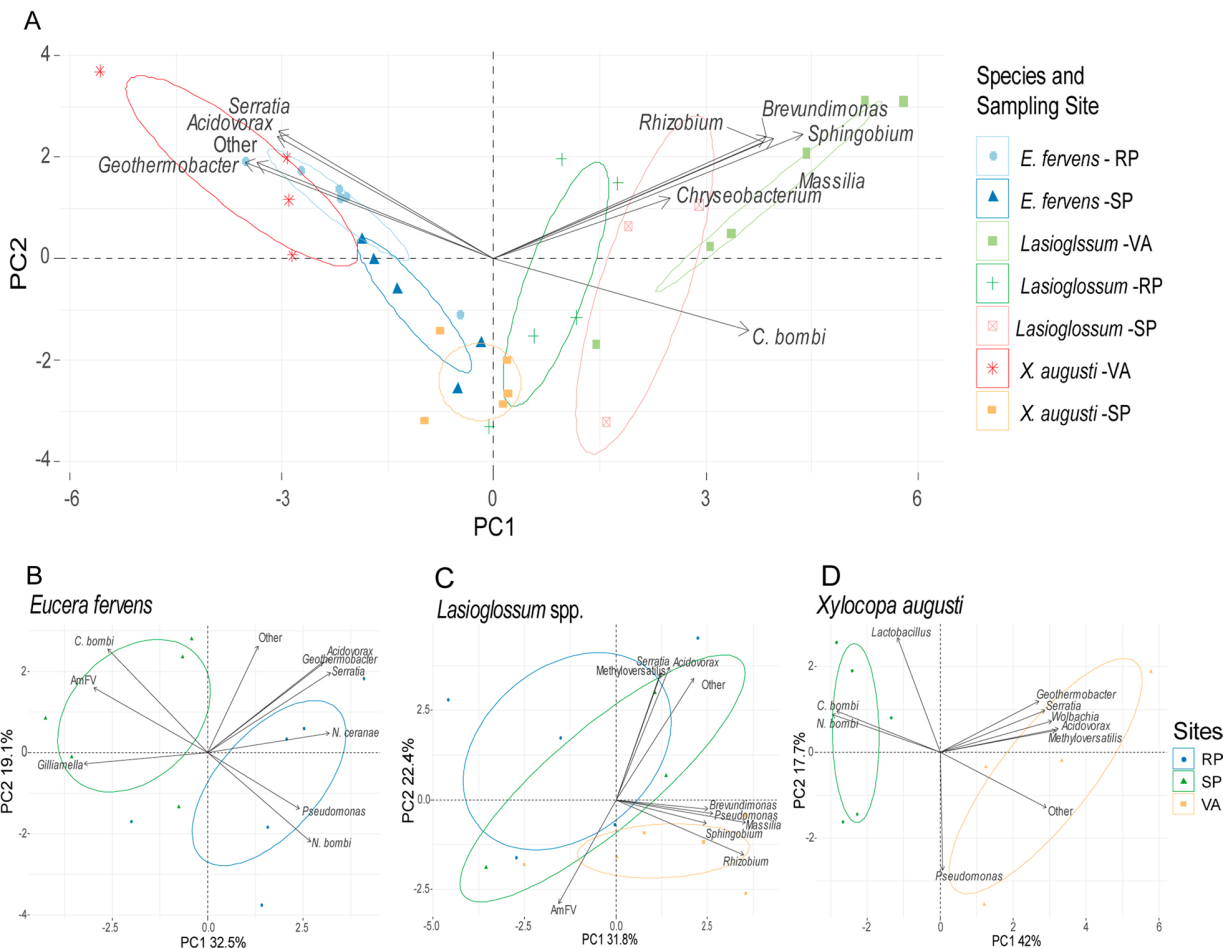
PCA analysis. **A** PCA was performed with 21 taxa at genus level; confidence ellipses are shown in the graph. The graph includes the top ten variables with the highest contribution. RP: Reserva Natural Paititi; SP: Santa Paula; VA: Vivero Antoniucci. **B** PCA considering *E. fervens* and the sampling sites RP and SP. **C** PCA considering *Lasioglossum* spp., and the sampling sites RP, SP and VA. **D** PCA considering *X. augusti*, and the sampling sites SP and VA

As a resume, the Principal Component Analysis (PCA) analysis showed the synergy between the land use, pathogens, and bee species (Fig. 5). Pathogens were found as the drivers of the gut microbiome shaping, especially affecting abundance of minor microbial groups in the gut microbiome and in the different sampling sites.

## Discussion

In this study the gut microbiome of three important solitary bees of South America (*X. augusti*, *E. fervens* and *Lasioglossum*) was studied, also examining the presence and load of potential gut pathogens and evaluating the effect of land use on microbiota and pathogen composition.

### Pathogen distribution in solitary bees

This research showed a wide and homogeneous spread of *N. ceranae* in all the wild pollinators sampled. The role of *A. mellifera* as a vector of a relevant number of pathogens, including *N. ceranae*, to wild bees has been described by Graystock et al. [52] and Furst et al. [53]. Since *A. mellifera* was recorded in all sampling sites with a high abundance, this could explain the spread of *N. ceranae* spores to flowers and consequently to local pollinators [54]. The homogeneity of *N. ceranae* on the wild pollinators sampled in the different territories is not surprising, also considering that *N. ceranae* spillover was defined as a new pandemic in pollinators [55]. Concerning *N. bombi*, this work reported its presence in all the studied bee species. Only few data are available on *N. bombi* spillover from the social bee *Bombus* spp. to other wild solitary bees. Differently from our results, the only other work investigating the *N. bombi* spread from *Bombus* to *Xylocopa* spp. did not detect its presence in *X. augusti* in Chile [56], probably due to the recent introduction of this solitary bee in Chile. The pathogen *C. bombi* seems to be affected by both the land use and the bee species. A similar result was found for *N. ceranae* in the study of Theodorou et al. [34], which showed that land use changes affected the nutritional resources correlated with the pathogen. In the Natural Reserve Paititi the recorded presence of *Bombus* spp. was twice when compared with the other sampling areas (data not shown in this manuscript). *Bombus pauloensis* is the most widespread native bee of the Buenos Aires province [57], and the ideal carrier of *C. bombi*, thus explaining the higher abundance of *C. bombi* in *X. augusti* sampled in the same area. Even when flowers could act as potential vectors for *C. bombi,* this occasionally happened with *E. fervens* since it is a highly specialized pollinator of pumpkins (e.g. *Cucurbita maxima*) [58]*.* It has already been highlighted that nutrition diversity impacts the load of *C. bombi* and the response of the solitary bees to this pathogen [59]. This may explain why *E. fervens*, having a diversified diet with respect to other solitary bees, has a lower load of *C. bombi* in comparison to *Lasioglossum* that feeds on a more diversified floral resources [60]*.*

### The peculiar gut microbiome of solitary bees

The gut microbiome of honeybees (*Apis mellifera*) was one of the first insect microbiome studied with NGS approaches, and it was proposed as a model for microbiome research [61]. The great attention and interest raised by the honeybee microbiome has led to a detailed knowledge on its acquirement, diversity, shaping by external factors [45, 62–64], and on its function in relation to the brain-gut axis [42, 65]. On the other hand, less is known for other pollinators and especially for solitary bees probably because of the difficulties in collection and study. The few studies available on non-social bee species showed a more variable and less distinctive microbiome if compared to social bees [66–68]. Solitary bees are also usually prone to maternally transmitted bacteria [69] and the acquisition of a homogeneous gut microbiome is presumably harder, therefore complicating the determination of the core taxa inhabiting the gut. An interesting debate on the definition of core taxa is still ongoing: Ainsworth et al. [70] described the core microbiome as the microbial taxa that occur with a frequency between 30 and 95% of relative abundance. Risely [71], on the other hand, gave less importance on the intensity factor and rather considered prevalence as the most important factor. For instance, in honey bees, the genus *Bifidobacterium* is considered a core microbial taxa despite being often found at low relative abundance (2–5%). For this reason, in the attempt to describe the core microbiota of the analyzed solitary bees, the abundance of *Bifidobacterium* in honey bees was taken as a reference threshold, in this study we focused our attention on the microbial genera with an equivalent or higher prevalence.

In this study we sampled *Lasioglossum* solitary bees, although it is known that some species of *Lasioglossum* show an incipient of social behavior. The two major microbial groups in the gut microbiome of *Lasioglossum* were *Lactobacillus* and *Sodalis* in accordance with Mayr et al., [72] and Rubin et al., [73]. *Lactobacillaceae* is an important and frequently found family in solitary and social bees responsible for the biosynthesis of some vitamins (*e.g.*: riboflavin and thiamin), amino acids [38] and short chain fatty acids, positively impacting social behavior and learning of the bees [65]. The majority of the NGS reads could be taxonomically assigned at genus level but not at species level, and possibly *Lasioglossum* is a reservoir of novel *Lactobacillus* species different from those isolated from *Apis* and *Bombus* spp. *Sodalis* is a microbial taxon widely present in the gut of solitary bees like *Megachile rotundata* [74], *Osmia* spp. and *Lasioglossum* [69] rather than social bees where it is present in relative low proportions [73]. *Sodalis* is a maternally transmitted microbial taxon in solitary bees [69] and this taxon showed differences in abundances among *Lasioglossum* species with different social behaviors, with higher abundance in species with a lower social behavior [73]. According to these findings, a very high relative abundance was found in the solitary *Lasioglossum* studied in this work. *Sodalis* might be an obligate endosymbiont for *Lasioglossum* as it is for other insects [75]. While other symbionts like *Gilliamella apicola* and *Snodgrassella alvi* have shown a negative correlation with *C. bombi* [76], our results demonstrated a *Sodalis* positive correlation with this Trypanosomatidae. Similar correlations between *Sodalis* and other Trypanosomatids like mutualistic *Trypanosoma brucei rhodesiense* have been reported in the literature for the Tse-tse fly *Glossina* spp. [77]*.*

In *Lasioglossum* the gut microbiome also showed a strong prevalence of *Pseudomonas* and *Wolbachia.* The co-occurrence of these two genera was also described in other insect’s gut microbiome like the cricket *Gryllus veletis* [78], although *Pseudomonas* was considered as a probable pathogen that varied its proportion with seasonality and according to *Wolbachia*´s abundance. Interestingly, Ge et al., [79] found similar proportion of *Pseudomonas* and *Wolbachia* in the reproductive system of *Paederus fuscipes* (coleoptera)*,* where *Pseudomonas* was found to contribute to the defense from predators thanks to the production of a toxic compound (Pederin), without decreasing the performance of the hosts. Moreover, *Wolbachia* was found to cope with adverse conditions triggered by *Pseudomonas*. The role of *Wolbachia* in the defense of the host from viral and bacterial pathogens is a recognized trait in different classes of insects [80, 81].

*E. fervens* gut microbiota has never been described despite its well-recognized importance in the pollination of some commercial crops in South America. Here, a strong presence of *Pseudomonas* was detected as occurred with *Lasioglossum* and *X. augusti*, and this is discussed below. Spiroplasma, the second most abundant taxa in *E. fervens,* is a widely found bacterium in ground beetles like *Pseudophonus* [82]. *Spiroplasma* species have been shown to protect the host by increasing defenses against pathogens [83] and it directly interacts with secreted antimicrobial molecules [84]. In fact, in this work *Spiroplasma* presence negatively correlated with *C. bombi* load, confirming this genus as a potential enhancer of the defense capabilities of the gut microbiome. Finally, *Gilliamella* and *Lactobacillus* were also detected with a high relative abundance. In honey bees, *Gilliamella* is the main responsible for pollen degradation together with *Lactobacillus*. In fact, the *Gilliamella* genus is reported to possess a complete metabolic pathway for pectin and hemicellulose degradation [38, 61]. *E. fervens* is well known to nourish mainly on pumpkin pollen [85], therefore this rich diet may justify the presence of *Gilliamella*. To the best of our knowledge, just one report on the gut microbiome of an *Eucera* species, different from *E. fervens*, was published to date [86]. Shapiro et al. [86] investigated the gut microbiome at order level of *Eucera pruinosa* and showed a major presence of Lactobacillales, Pseumonadales and Cytophagales that broadly reflects our findings.

The latest reports on the *Xylocopa* spp. gut microbiome vary considerably with *Xylocopa* species and the different sampling sites. Holley et al. [87], found a very high proportion of *Bombiscardovia*, *Pseudomonas*, *Xenorhabdus,* and different Lactobocillaceae genera (*Apilactobacillus*, *Bombilactobacillus*, *Lactobacillus* and *Latilactobacillus*) in *Xylocopa micans* and *Xylocopa tabaniformis*. Differently, Handy et al. [88], studied *Xylocopa sonorina* and *Xylocopa tabaniformis* sampled from California and Arizona detected *Apibacter*, *Schmidhempelia*, *Enteromonas*, *Enterobacter* and *Fructobacillus* as the main genera. The results shown by Handy et al. [88], supported the influence of the landscape on the gut microbiome. Our results did not support the influence of land use on the gut microbiome of *X. augusti*, nevertheless the differences in the gut microbiome profiles obtained in this work, when compared to Handy et al. [88] and Holley et al. [87], could be explained both by the different land use and species considered. However, in our case, within Lactobacillaceae, only the genus *Lactobacillus* was detected, differently from Holley et al. [87]. In our study, *Apilactobacillus*, *Bobilactobacillus* and *Latilactobacillus* were below the limit of detection, perhaps due to the impact of land use on the gut microbiome. This is supported by the fact that a relatively low proportion of Lactobacilaceae was also detected in *Lasioglossum* and *E. fervens* in the same conditions and sampling period. *Pseudomonas* was frequently found in the *Xylocopa* individuals sampled by Holley et al. [88] in Texas but not in Handy et al. [88] samples obtained in California and Arizona. This confirms that gut microbiome acquisition is strongly dependent on the environmental conditions.

### *Pseudomonas* as the major group in solitary bees

The role of *Pseudomonas* in the gut microbial communities of the solitary bees analyzed in this study is not well understood yet, but in social bees the presence of *Pseudomonas* seems to be correlated with the presence of molecules of anthropic origin such as the antibiotic tylosin [45] or more probably to bees collecting water where *Pseudomonas* is widely diffused [89]. A possible explanation of the high *Pseudomonas* abundance in the analyzed bees within this study may be the extensive use of glyphosate in the area from which bees were sampled. In the last three decades, in the Pampas region, the use of glyphosate has increased because of the spread of glyphosate resistant crops [90]. It is well known that *Pseudomonas* can catabolize this molecule and use it as additional carbon source [91–93] and, therefore, its abundance may be an adaptation to contaminated nectar. Indeed, a remarkable amount of honey samples resulted to be contaminated with glyphosate worldwide [94, 95] but also in the sampling district within the Pampas of this work [96]. Recently, Motta et al., [96] showed that glyphosate can perturb the gut microbiome of honey bees, but the perturbation might also be an adaptation to the xenobiotics. To confirm the role of insect gut bacteria in xenobiotic degradation, it has been observed that in the wasp *Nasonia vitripennis* both the gut bacteria *Serratia* and *Pseudomonas* contributed to atrazine degradation, conferring resistance to wasp populations [93].

## Conclusion

This work supports the hypothesis that land use and anthropization pressure contribute to weakening bee health predisposing bees to pathogens proliferation. Anthropization itself contributes to biotic stressors spread and to biotic and abiotic stressors incidence, which can impact on the microbiota composition.  Although there was a trend towards significant variation in the gut microbial composition of native bees, our results did not show a direct correlation between land use and gut microbiota changes in solitary bees. However, land use influenced the presence and load of pathogens, which are the main contributors to the shaping of the gut microbiota. Therefore, the microbiota changes can be an indirect effect of land use caused by decreased nutrient sources and water availability, and by the human mediated spread of social pollinators. Additionally, this study characterized for the first time the core microbiome of wild pollinators of Argentina, contributing to the knowledge on the solitary bee microbiome composition. Nevertheless, the current knowledge does not allow a clear understanding whether the microbiome changes can positively or negatively influence the host growth and health, and consequently the life span of studied solitary pollinators.

## Supplementary Information

**Additional file 1.** Supplementary materials.

## Data Availability

NGS raw sequence data have been submitted to NCBI repository under the Sequence Read Archive (SRA) databases under the Bioproject N° PRJNA799463. Accession numbers SAMN25173657—SAMN25173686 belong to *Lasioglossum* samples; SAMN25173642—SAMN25173656 belong to *Eucera fervens* samples*;* SAMN25173688—SAMN25173697 belong to *Xylocopa augusti* samples. The datasets generated and/or analyzed during the current study are available in the Mendelay Data repository: Published: 12 December 2022 |Version 1| https://doi.org/10.17632/jrs7rr8vw5.1.
