# Supplementary material for: The gut microbiome of solitary bees is mainly affected by pathogen assemblage and partially by land use
Source: Environ Microbiome. 2023 Apr 26;18:38. doi: 10.1186/s40793-023-00494-w (PMC10131457; doi:10.1186/s40793-023-00494-w)
Supplement: Supplementary file 1 — Additional file 1. Supplementary materials. [file 40793_2023_494_MOESM1_ESM.pdf]

Supplementary materials for:

The gut microbiome of solitary bees is mainly affected by pathogen assemblage and partially by land use

CONICET: Gregorio Fernandez De Landa[1,2], Daniele Alberoni[3]*, Loredana Baffoni[3], Mateo Fernandez de Landa[1,2], Pablo Damian Revainera[1,2], Leonardo Pablo Porrini[1,2], Constanza Brasesco[1,2], Silvina Quintana[1,2], Francisco Zumpano[4], Martin Javier Eguaras[1,2], Matias Daniel Maggi[1,2], Diana Di Gioia[3].

[1]Instituto de Investigaciones en Producción Sanidad y Ambiente (IIPROSAM), Facultad de Ciencias Exactas y Naturales, Universidad Nacional de Mar del Plata, Centro Científico Tecnológico Mar del Plata, CONICET. Centro de Asociación Simple CIC PBA, Mar del Plata, Argentina.

[2]Centro de Investigaciones en Abejas Sociales, Facultad de Ciencias Exactas y Naturales, Universidad Nacional de Mar del Plata, Mar del Plata, Argentina.

[3]Dipartimento di Scienze e Tecnologie Agro-Alimentari, University of Bologna, Viale Fanin 44, 40127, Bologna, Italy;

[4]Instituto de Investigaciones Marinas y Costeras (IIMyC), Facultad de Ciencias Exactas y Naturales, Universidad Nacional de Mar del Plata-CONICET. Funes 3350 Mar del Plata (7600), Argentina

[*] Corresponding Author:

Daniele Alberoni: daniele.alberoni@unibo.it

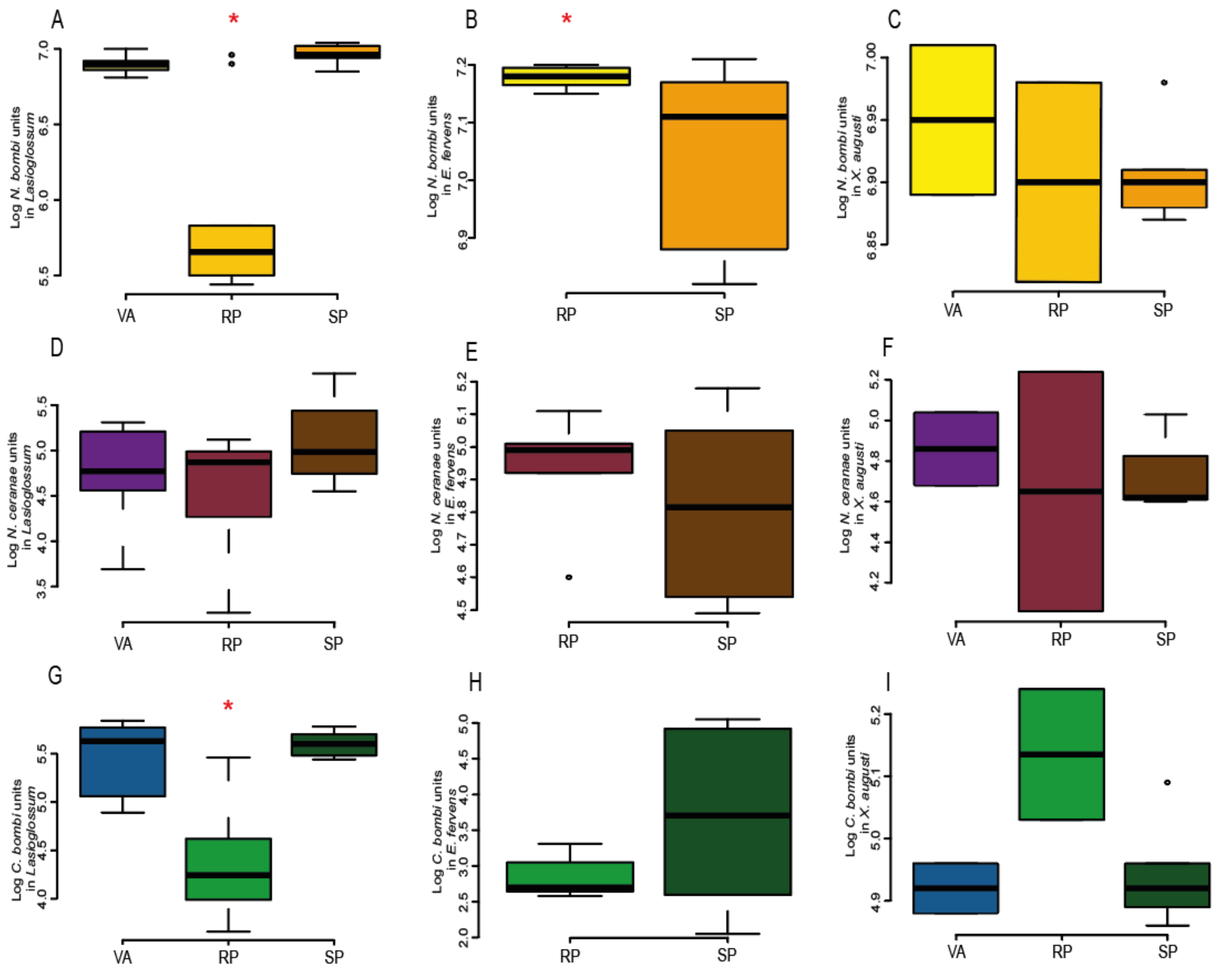

Fig S1A–S1I *Boxplot describing the pathogens counts per sampling site and solitary bee species.* qPCR results for the gut pathogen *Nosema bombi* expressed as *N. bombi* Units (NbU) per bee: **A)** in *Lasioglossum* per sampling site; **B)** in *E. fervens* per sampling site; **C)** in *X. augusti* per sampling site. qPCR results for the gut pathogen *Nosema cerane* expressed as *N. ceranae* Units (NcU) per bee: **D)** in *Lasioglossum* per sampling site; **E)** in *E. fervens* per sampling site; **F)** in *X. augusti* per sampling site. qPCR results for the pathogen *Crithidia bombi* expressed as *C. bombi* Units (CbU) per bee: **G)** in *Lasioglossum* per sampling site; **H)** in *E. fervens* per sampling site; **I)** in *X. augusti* per sampling site. The sampling sites: VA - Vivero Antoniucci; RP - Reserva Natual Paititi; SP - Santa Paula Farm. *<0.05

Table S1. Primer list. Table with complete list of primers used for the PCR and qPCR

PATHOGEN	PRIMER	SECUENCE (5' - 3')	MELTING TEMPERATURE	ANILING TEMPERATURE	AMPLICON SIZE	CITE
16S rRNA gene (V6-V8)	799F	TCGTCGGCAGCGTCAGATGTGTATAAGAGACAGAACMGGATTAGATACCCKG	N.A	60	470 and 720	46
	1139R	GTCTCGTGGGCTCGGAGATGTGTATAAGAGACAGACGTCATCCCCACCTTCC				
Lotmaria passim	Lp2F 459	AGGGATATTTAAACCCATCGAA		60	459	97
	Lp2R 459	ACCACAAGAGTACGGAATGC				
Ascophaera spp.	AscoAll1	GCACTCCCACCCTTGTCT A	89.8	58	550	98
	AscoAll2	GAWCACGACGCCGTCACT				
Nosema apis	321 apis fw	GGGGGCATGTCTTTGACGTACTATGTA	84.3	60	321	99
	321 apis rv	GGGGGGCGTTTAAAATGTGAAACAACTATG				
Nosema cerenae	218 mitoc fw	CGGCGACGATGTGATATGAAAATATTAA	84.3	60	218	99
	218 mitoc rv	CCCGGTCATTCTCAAACAAAAAACCG				
Apicystis bombi	ApBF1 293 fw	CGTACTGCCCTGAATACTCCAG	81.7	58	293	32
	ApBR1 293 rv	TGAAAGCGGCGTATACATGA				
Crithidia bombi	Crithidia 119f	CCAACGGTGAGCCGCATTCAGT	83.7	58	119	100
	crithidia 119 rv	CGCGTGTCGCCCAGAACATTGA				
Nosema bombi	NBOMBI 323 fw	CCATGCATGTTTTTGAAGATTATTAT	83.5	56	323	101
	NBOMBI 323 rv	CATATATTTTTAAAATATGAAACAATAA				
Apis mellifera Filamentous Virus	AmFV 551 -F	CAGAGAATTCGGTTTTTGTGAGTG	84.7	52	551	102
	AmFV 551 -R	CATGGTGGCCAAGTCTTGCT				
Internal Control	Control Interno Apidae-F	AGATGGGGGCATTCGTATTG	84.5	60		32
	Control Interno Apidae-R	ATCTGATCGCCTTCGAACCT				

Table S2: Samples information

Native Bee sampled								
**Sample name**	**Bee species**	**Sampling date**	**Sampling place**	**Season**	**Total DNA from the bees (ng)**	**Total *Nosema bombi* Spores**	**Total *Nosema ceranae* Spores**	**Total *Critidia bombi***
las-001	*Lasioglossum spp.*	03-09-2020	*Reserva Natural Paititi*	Summer	28.5	0	2.33E+05	2.87E+04
las-002	*Lasioglossum spp.*	03-09-2020	*Reserva Natural Paititi*	Summer	32.65	7.88E+05	3.35E+05	1.44E+04
las-003	*Lasioglossum spp.*	03-09-2020	*Reserva Natural Paititi*	Summer	46	9.05E+05	3.62E+05	3.61E+03
las-004	*Lasioglossum spp.*	03-09-2020	*Reserva Natural Paititi*	Summer	563.5	4.35E+05	6.76E+05	8.55E+03
las-005	*Lasioglossum spp.*	02-12-2019	*Reserva Natural Paititi*	Summer	22.45	9.95E+04	3.28E+05	1.47E+04
las-006	*Lasioglossum spp.*	02-12-2019	*Reserva Natural Paititi*	Summer	43.35	0.00E+00	3.32E+05	4.00E+04
las-007	*Lasioglossum spp.*	02-12-2019	*Reserva Natural Paititi*	Summer	47.35	6.82E+05	5.41E+05	8.06E+03
las-008	*Lasioglossum spp.*	02-12-2019	*Reserva Natural Paititi*	Summer	47.35	1.13E+06	5.48E+05	9.24E+03
las-009	*Lasioglossum spp.*	02-12-2019	*Reserva Natural Paititi*	Summer	27.85	2.60E+05	1.20E+07	2.46E+05
las-010	*Lasioglossum spp.*	02-12-2019	*Reserva Natural Paititi*	Summer	7.55	1.38E+04	3.96E+06	2.87E+05
las-011	*Lasioglossum spp.*	04-05-2019	*Santa Paula´s Farm*	Autumn	35.35	0	9.19E+06	3.88E+05
las-012	*Lasioglossum spp.*	04-05-2019	*Santa Paula´s Farm*	Autumn	22.4	0	8.16E+06	2.62E+05
las-013	*Lasioglossum spp.*	04-05-2019	*Santa Paula´s Farm*	Autumn	63.5	0	7.84E+06	4.43E+05
las-014	*Lasioglossum spp.*	02-16-2020	*Santa Paula´s Farm*	Summer	18.1	3.03E+05	8.08E+06	3.90E+05
las-015	*Lasioglossum spp.*	02-16-2020	*Santa Paula´s Farm*	Summer	57.5	9.14E+05	1.13E+07	3.67E+05
las-016	*Lasioglossum spp.*	02-16-2020	*Santa Paula´s Farm*	Summer	55	6.13E+06	1.01E+07	4.04E+05
las-017	*Lasioglossum spp.*	02-16-2020	*Santa Paula´s Farm*	Summer	70.5	7.51E+05	7.53E+06	2.61E+05
las-018	*Lasioglossum spp.*	02-12-2019	*Santa Paula´s Farm*	Summer	27.6	0	8.56E+06	5.57E+05
las-019	*Lasioglossum spp.*	02-12-2019	*Santa Paula´s Farm*	Summer	45.35	0	8.48E+06	5.12E+05
las-020	*Lasioglossum spp.*	02-12-2019	*Santa Paula´s Farm*	Summer	28.8	0	7.77E+06	4.66E+05
las-021	*Lasioglossum spp.*	01-16-2020	*Vivero Antoniucci*	Summer	34	4.78E+05	9.26E+06	3.67E+05
las-022	*Lasioglossum spp.*	01-16-2020	*Vivero Antoniucci*	Summer	25.2	2.99E+05	6.94E+06	3.36E+05
las-023	*Lasioglossum spp.*	01-16-2020	*Vivero Antoniucci*	Summer	28.1	1.16E+05	8.37E+06	4.81E+05
las-024	*Lasioglossum spp.*	12-05-2019	*Vivero Antoniucci*	Spring	82	7.40E+05	7.25E+06	6.64E+05
las-025	*Lasioglossum spp.*	12-05-2019	*Vivero Antoniucci*	Spring	24.45	1.65E+06	6.65E+06	5.86E+05
las-026	*Lasioglossum spp.*	12-05-2019	*Vivero Antoniucci*	Spring	62	2.89E+05	8.61E+06	5.76E+05
las-027	*Lasioglossum spp.*	03-15-2019	*Vivero Antoniucci*	Summer	60	0	7.05E+06	5.07E+05
las-028	*Lasioglossum spp.*	03-15-2019	*Vivero Antoniucci*	Summer	17.75	0	6.83E+06	1.30E+05
las-029	*Lasioglossum spp.*	03-15-2019	*Vivero Antoniucci*	Summer	56.5	1.31E+06	7.91E+06	7.82E+04
las-030	*Lasioglossum spp.*	03-15-2019	*Vivero Antoniucci*	Summer	22.05	9.80E+05	7.57E+06	8.20E+04
xyl-001	*Xylocopa augusti*	04-05-2019	*Santa Paula´s Farm*	Autumn	96.5	9.47E+05	7.84E+06	6.94E+04
xyl-002	*Xylocopa augusti*	04-05-2019	*Santa Paula´s Farm*	Autumn	29.25	4.36E+05	8.24E+06	1.28E+05

xyl-003	*Xylocopa augusti*	04-05-2019	*Santa Paula´s Farm*	Autumn	24.85	0	6.90E+06	9.49E+04
xyl-004	*Xylocopa augusti*	02-16-2020	*Santa Paula´s Farm*	Summer	496.5	0	9.06E+06	8.13E+04
xyl-005	*Xylocopa augusti*	02-16-2020	*Santa Paula´s Farm*	Summer	66.5	6.97E+05	7.67E+06	7.93E+04
xyl-006	*Xylocopa augusti*	03-09-2020	*Reserva Natural Paititi*	Summer	22.65	7.58E+05	7.32E+06	1.60E+05
xyl-007	*Xylocopa augusti*	03-09-2020	*Reserva Natural Paititi*	Summer	29.95	1.28E+06	7.56E+06	1.06E+05
xyl-008	*Xylocopa augusti*	05-12-2019	*Vivero Antoniucci*	Spring	15.55	1.52E+06	8.04E+06	1.10E+05
xyl-009	*Xylocopa augusti*	05-12-2019	*Vivero Antoniucci*	Spring	48.65	9.05E+05	7.79E+06	8.48E+04
euc-001	*Eucera fervens*	02-16-2020	*Santa Paula´s Farm*	Summer	15	7.72E+05	9.77E+06	2.10E+05
euc-002	*Eucera fervens*	02-16-2020	*Santa Paula´s Farm*	Summer	7.25	9.18E+05	7.92E+06	8.11E+04
euc-003	*Eucera fervens*	02-16-2020	*Santa Paula´s Farm*	Summer	0	0	7.44E+06	9.14E+04
euc-004	*Eucera fervens*	02-16-2020	*Santa Paula´s Farm*	Summer	16.85	0	6.76E+06	9.74E+04
euc-005	*Eucera fervens*	02-16-2020	*Santa Paula´s Farm*	Summer	6.7	3.73E+05	1.39E+07	3.24E+04
euc-006	*Eucera fervens*	02-16-2020	*Santa Paula´s Farm*	Summer	17.15	6.37E+05	1.56E+07	6.21E+02
euc-007	*Eucera fervens*	02-16-2020	*Santa Paula´s Farm*	Summer	188	0	1.38E+07	7.62E+02
euc-008	*Eucera fervens*	02-16-2020	*Santa Paula´s Farm*	Summer	2.85	4.75E+05	1.43E+07	3.93E+02
euc-009	*Eucera fervens*	02-16-2020	*Santa Paula´s Farm*	Summer	32.95	0.00E+00	1.53E+07	3.33E+02
euc-010	*Eucera fervens*	02-16-2020	*Santa Paula´s Farm*	Summer	27.45	7.92E+05	1.43E+07	2.19E+02
euc-011	*Eucera fervens*	03-09-2020	*Reserva Natural Paititi*	Summer	34.65	0	1.49E+07	6.92E+02
euc-012	*Eucera fervens*	03-09-2020	*Reserva Natural Paititi*	Summer	6.3	5.70E+05	1.43E+07	3.78E+02
euc-013	*Eucera fervens*	03-09-2020	*Reserva Natural Paititi*	Summer	27.8	2.03E+05	1.51E+07	1.03E+03
euc-014	*Eucera fervens*	02-03-2019	*Reserva Natural Paititi*	Summer	29.95	8.86E+05	1.47E+07	3.28E+02
euc-015	*Eucera fervens*	02-03-2019	*Reserva Natural Paititi*	Summer	46.65	3.22E+05	1.41E+07	7.03E+02
euc-016	*Eucera fervens*	02-03-2019	*Reserva Natural Paititi*	Summer	4.65	3.88E+05	1.29E+07	2.29E+02
euc-017	*Eucera fervens*	02-03-2019	*Reserva Natural Paititi*	Summer	13.65	0	1.51E+07	3.15E+02

Table S3. Sampling Site Land Use. The table summarizes the different land uses at each sampling location. "Category" refers to the division provided by the urBAsig repositories (https://www.urbasig.gob.gba.gob.ar/), while “Land Use” refers to a subdivision of "Category". Finally, the percentage is calculated for each study site individually.

LOCATION	CATEGORY	LAND USE	AREA km²	PERCENTAGE
ANTONIUCCI	INTENSIVE EXTRACTIVE ACTIVITY	INTENSIVE FARMING	20.25420699	78.698
	INTENSIVE EXTRACTIVE ACTIVITY	PLANT NURSERY	1.2076484	4.392194913
	RECREATIONAL CENTER	RECREATIONAL SPACES	0.156483722	0.569128405
	URBAN RESERVE	BIOLOGICAL CORRIDORS	1.294178724	4.706904103
	PARKS & GREEN SPACES	RECREATIONAL OPEN SPACES	0.055657681	0.202425957
	PARKS & GREEN SPACES	CEMETERY	0.288009639	1.047485735
	RESIDENCIAL (HIGH-DENSITY)	HOUSING AND COMMERCIAL	4.229451314	15.38243627
SANTA PAULA	INTENSIVE EXTRACTIVE ACTIVITY	INTENSIVE FARMING	25.86421543	91.52579227
	EXTENSIVE EXTRACTIVE ACTIVITY	EXTENSIVE FARMING	1.645446854	5.822748708
	NATURAL RESERVE	LAGOON	0.267021986	0.944911664
	INDUSTRIAL	INDUSTRIAL	0.111924005	0.39606588
	BIOLOGICAL CORRIDORS	BIOLOGICAL CORRIDORS	0.098200652	0.347503001
	RESIDENTIAL (LOW-DENSITY)	HOUSING AND COMMERCIAL	0.271166013	0.959576147
	RECREATIONAL CENTER	HYPODROME	0.000961462	0.003402329
RESERVA NATURAL PAITITI	EXTENSIVE EXTRACTIVE ACTIVITY	EXTENSIVE FARMING	13.92698064	49.28541599
	INTENSIVE EXTRACTIVE ACTIVITY	INTENSIVE FARMING	8.884006057	31.43911415
	RESIDENTIAL (LOW-DENSITY)	HOUSING AND COMMERCIAL	0.147974946	0.523660293
	NATURAL RESERVE	RESERVE	5.298851262	18.75180956

Table S4: α-diversity output per sampling site

α-diversity index	Group1	Group2	Group1 mean	Group1 std	Group2 mean	Group2 std	t stat	*p*-value
				Xylocopa augusti				
**Chao1**	Santa Paula	Antoniucci	11.802	2.252	13.813	1.523	-1	0,205
**Observed OTUs**	Santa Paula	Antoniucci	4946,400	976,625	5530,520	489,096	-1,029	0,346
**PD whole tree**	Santa Paula	Antoniucci	247,424	47,122	293,308	25,385	-1,645	0,128
				Eucera fervens				
**Chao1**	Paititi	Santa Paula	16941,735	998,086	18454,861	1815,859	-1,582	0,133
**Observed OTUs**	Paititi	Santa Paula	6371,833	471,635	6161,020	493,224	0,654	0,541
**PD whole tree**	Paititi	Santa Paula	367,473	28,047	346,200	23,450	1,219	0,250
				Lassioglossum				
**Chao1**	Paititi	Santa Paula	18980,096	5082,403	19542,057	8007,260	-0,105	1
	Paititi	Antoniucci	18980,096	5082,403	23566,843	2563,447	-1,750	0,399
	Santa Paula	Antoniucci	19542,057	8007,260	23566,843	2563,447	-0,989	1
**Observed OTUs**	Paititi	Santa Paula	6814,680	1755,229	6313,267	2616,545	0,281	1
	Paititi	Antoniucci	6814,680	1755,229	7537,317	1217,758	-0,726	1
	Santa Paula	Antoniucci	6313,267	2616,545	7537,317	1217,758	-0,844	1
**PD whole tree**	Paititi	Santa Paula	412,071	97,363	409,113	161,446	0,028	1
	Paititi	Antoniucci	412,071	97,363	481,406	73,642	-1,215	0,660
	Santa Paula	Antoniucci	409,113	161,446	481,406	73,642	-0,813	1

Table S5: β-diversity output per sampling site

Group 1	Group 2	*p*	*Bee*
weighted UniFrac			
Paititi *vs* Paititi	Santa_Paula *vs* Santa_Paula	0.02	***E. fervens***
Paititi *vs* Paititi	Santa Paula *vs* Santa Paula	0.74	***Lassioglossum***
Paititi *vs* Paititi	Antoniucci *vs* Antoniucci	0.07	***Lassioglossum***
Antoniucci *vs* Antoniucci	Santa Paula *vs* Santa Paula	0.14	***Lassioglossum***
Antoniucci *vs* Antoniucci	Santa Paula *vs* Santa Paula	0.04	***X. augusti***
unweighted UniFrac			
Paititi *vs* Paititi	Santa Paula *vs* Santa Paula	0.07	***E. fervens***
Paititi *vs* Paititi	Santa Paula *vs* Santa Paula	1.00	***Lassioglossum***
Paititi *vs* Paititi	Antoniucci *vs* Antoniucci	1.00	***Lassioglossum***
Antoniucci *vs* Antoniucci	Santa Paula *vs* Santa Paula	1.00	***Lassioglossum***
Antoniucci *vs* Antoniucci	Santa Paula *vs* Santa Paula	0.10	***X. augusti***

References

97. Arismendi N, Bruna A, Zapata N, Vargas M. PCR-specific detection of recently described Lotmaria passim (Trypanosomatidae) in Chilean apiaries. J. Invertebr. Pathol. 2016;134:1-5.

98. James RR, Skinner JS. PCR diagnostic methods for Ascosphaera infections in bees. J. Invertebr. Pathol. 2005;90(2):98-103.

99. Martín-Hernández R, Meana A, Prieto L, Salvador AM, Garrido-Bailón E, Higes M. Outcome of colonization of Apis mellifera by Nosema ceranae. Appl. Environ. Microbiol. 207;73(20):6331-6338.

100. Huang WF, Skyrm K, Ruiter R, Solter L. Disease management in commercial bumble bee mass rearing, using production methods, multiplex PCR detection techniques, and regulatory assessment. Journal of Apicultural Research, 2015;54(5):516-524.

101. Klee J, Tay WT, Paxton RJ. Specific and sensitive detection of Nosema bombi (Microsporidia: Nosematidae) in bumble bees (Bombus spp.; Hymenoptera: Apidae) by PCR of partial rRNA gene sequences. J. Invertebr. Pathol. 2006;91(2):98-104.

102. Hartmann U, Forsgren E, Charriere JD, Neumann P, Gauthier L. Dinamica delle infezioni da Apis mellifera filamentous virus (AmFV) nelle api mellifere e relazioni con altri parassiti. Virus. 2015;7(5):2654-2667.
